# The Impact of Feature Extraction on Classification Accuracy Examined by Employing a Signal Transformer to Classify Hand Gestures Using Surface Electromyography Signals

**DOI:** 10.3390/s24041259

**Published:** 2024-02-16

**Authors:** Aly Medhat Moslhi, Hesham H. Aly, Medhat ElMessiery

**Affiliations:** 1 Faculty of Engineering, The Arab Academy for Science, Technology & Maritime Transport, Smart Village Campus, Giza P.O. Box 2033, Egypt; hesham_aly@aast.edu; 2 Faculty of Engineering, Cairo University, Giza P.O. Box 2033, Egypt; messiery@eng.cu.edu.eg

**Keywords:** surface electromyography, EMG, transformer, feature extraction, hand gesture recognition

## Abstract

Interest in developing techniques for acquiring and decoding biological signals is on the rise in the research community. This interest spans various applications, with a particular focus on prosthetic control and rehabilitation, where achieving precise hand gesture recognition using surface electromyography signals is crucial due to the complexity and variability of surface electromyography data. Advanced signal processing and data analysis techniques are required to effectively extract meaningful information from these signals. In our study, we utilized three datasets: NinaPro Database 1, CapgMyo Database A, and CapgMyo Database B. These datasets were chosen for their open-source availability and established role in evaluating surface electromyography classifiers. Hand gesture recognition using surface electromyography signals draws inspiration from image classification algorithms, leading to the introduction and development of the Novel Signal Transformer. We systematically investigated two feature extraction techniques for surface electromyography signals: the Fast Fourier Transform and wavelet-based feature extraction. Our study demonstrated significant advancements in surface electromyography signal classification, particularly in the Ninapro database 1 and CapgMyo dataset A, surpassing existing results in the literature. The newly introduced Signal Transformer outperformed traditional Convolutional Neural Networks by excelling in capturing structural details and incorporating global information from image-like signals through robust basis functions. Additionally, the inclusion of an attention mechanism within the Signal Transformer highlighted the significance of electrode readings, improving classification accuracy. These findings underscore the potential of the Signal Transformer as a powerful tool for precise and effective surface electromyography signal classification, promising applications in prosthetic control and rehabilitation.

## 1. Introduction

Surface electromyography (sEMG) signals play a pivotal role in the determination of hand gestures. These signals are essentially the summation of motor action potentials generated beneath the skin during muscle contractions. sEMG signals hold great promise as an interface for discerning hand gestures and find various applications, particularly in the field of rehabilitation [1,2,3,4]. Rehabilitation primarily targets individuals coping with muscular, neurological, or osteoarticular disorders [5]. The monitoring and analysis of a patient’s physiological information during the rehabilitation process are of utmost importance, as this information encompasses both physical aspects, such as muscle force, and psychological elements, such as the patient’s intentions [6]. The accurate decoding of sEMG signals is essential to distinguish these aspects. Moreover, applications like sign language recognition [7] and human–computer interaction [8] also rely on precise decoding of sEMG signals [8].

One of the significant challenges associated with sEMG signals is their susceptibility to overfitting, especially when transitioning between different individuals. When classifiers trained on data from one person are applied to a new user, their performance tends to be only slightly better than random chance. Several factors contribute to the variability of sEMG signals between individuals, including body fat percentage [9], age [10], fatigue [11], sex, and external factors like power line interference [12] and electrode placement [13]. Consequently, effectively decoding sEMG signals necessitates the deployment of advanced detection, filtering, processing, and classification algorithms [14].

Typically, the challenge posed by significant variations between individuals is tackled as a classification problem. In this context, the classifier takes electrode data as inputs and produces an output corresponding to one of the recognized hand gestures (classes) [15,16,17]. The underlying idea involves extracting multidimensional features from the signals, rather than solely relying on amplitude, and employing data analysis and pattern recognition techniques to predict the intended gesture. Machine learning techniques, such as Support Vector Machine (SVM) [18] and random forest [19], often serve as the foundation for classification.

In this work, the power of Transformers is being utilized for the classification of densely packed signals. Transformers, originally designed for natural language processing, are being adapted for the task of signal classification by creating a novel method for signal classification referred to as “Signal Transformer (ST)”. By utilizing their attention mechanisms and deep neural network architecture, a robust and accurate classification model is being developed to handle complex signal data. This innovative approach has the potential to significantly improve the accuracy and efficiency of signal classification across various applications.

Our study delves into the realm of feature extraction and its impact on classification accuracy. To explore this, we investigate two distinct techniques for feature extraction from sEMG signals prior to classification. These techniques encompass the utilization of the Fast Fourier Transform (FFT) wavelet extraction for feature extraction. The FFT is an algorithm that efficiently computes the discrete Fourier transform of a sequence, significantly speeding up the process of analyzing frequencies within a signal [20].

In this research, the newly introduced preprocessing phase plays a pivotal role in the effectiveness of the Signal Transformer model. A newly introduced preprocessing pipeline specifically tailored for sEMG signals was developed, involving advanced noise filtering, normalization techniques, and signal encoding processes. The Transformer model, traditionally used in natural language processing, was innovatively adapted to tackle the complex task of sEMG signal classification, leading to the creation of what is termed the “Signal Transformer”. This adaptation marks a significant departure from conventional Transformer applications, showcasing a unique approach. Key modifications included the development of a signal-specific preprocessing protocol; the integration of enhanced feature extraction layers designed for high-dimensional signal data; the adaptation of the input layer, initially suitable for embedding words in natural language processing tasks, to accept continuous signals generated from sEMG electrodes (bearing in mind that the number of electrodes varies from case to case, necessitating a fixed number of input parameters for the Transformer without data loss); the introduction of a signal embedding layer; the optimization of the overall model architecture to suit the high-frequency nature of bio-signals; and a tailored training approach addressing the stochastic characteristics of sEMG data. Collectively, these modifications transform the traditional Transformer model into a more robust and specialized framework for sEMG signal processing. The Signal Transformer not only demonstrates the potential to extend the boundaries of deep learning applications but also highlights the possibility of significant advancements in the field of bio-signal analysis.

## 2. Literature Review

Gesture recognition, including continuous gesture recognition and sign language gesture recognition, represents a significant area in computational linguistics and human–computer interaction. This field focuses on enabling machines to interpret human gestures as a means of communication or interaction. Continuous gesture recognition involves tracking and interpreting gestures in a fluid, uninterrupted manner, making it crucial for real-time applications. Sign language gesture recognition, on the other hand, is dedicated to translating sign language, used by the deaf and hard-of-hearing community, into text or speech. This area is vital for creating inclusive technologies that bridge communication gaps. Both tasks demand high accuracy and real-time processing capabilities to be effective [21].

The fundamental technique for capturing EMG signals involves either the insertion of intermuscular electrodes (invasive method) or the attachment of surface electrodes (non-invasive method) to the muscle under investigation, subsequently recording the signal [22].

**Figure 1:**
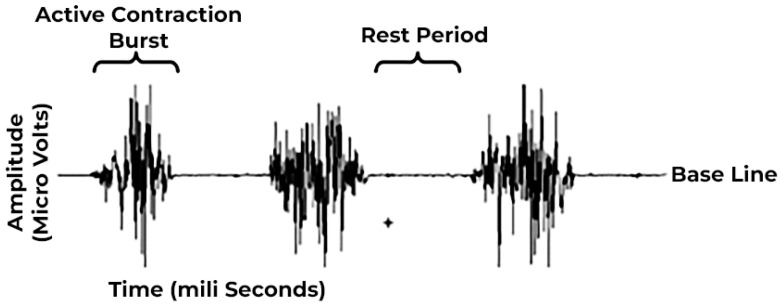
Raw EMG signals.

The EMG signal, depicted in Figure 1, exhibits a frequency range of 50–500 Hz [12] and manifests in two states: a steady state and a transient state during muscle activation. The steady-state EMG potential typically ranges around −80/−90 mV [12], whereas the contraction potential spans from −5 to 5 mV [14,23].

The term “decoding the sEMG” refers to a set of techniques and methodologies aimed at extracting data from activated skeletal muscles through physiological neural activity. This extracted information can be employed to control various devices, such as exoskeletons or prosthetic hands.

EMG signals, by their nature, exhibit complex and highly variable information. Extracting meaningful insights from these signals necessitates the application of advanced pattern recognition and data analysis techniques akin to those used in data analysis [24]. Recent studies on sEMG signal decoding revealed that these studies follow similar approaches, which can be summarized as follows: (1) signal acquisition, (2) preprocessing, (3) feature extraction, and (4) classification and evaluation.

### 2.1. Signal Acquisition

Despite the nonstationary characteristics of sEMG signals, they can still be detected using surface electrodes [25]. Electrodes are typically classified based on their type (gel-filled or dry electrodes) and density (linear or 2D array) [24]. The sensor used for sEMG acquisition should adhere to the Nyquist–Shannon theorem [26], ensuring a sampling frequency that is at least twice the highest frequency of sEMG signals, necessitating a sampling frequency greater than 1000 Hz.

### 2.2. Preprocessing

The challenge with raw sEMG data lies in the high noise captured during signal acquisition, requiring extensive processing for accurate signal decoding. There are primarily three types of noise in sEMG signals: (1) inherent noise from electronic components, (2) power frequency interference from the power system, and (3) noise originating from the electrodes [25]. Preprocessing, a crucial step before applying Machine Learning (ML) or deep learning (DL) techniques for sEMG decoding, significantly enhances subsequent performance. Preprocessing encompasses several key steps, including filtering, rectification, normalization, and segmentation.

#### 2.2.1. Filtering

Filtering is essential to reduce artifacts in the sEMG signals. In some studies, both a Band pass filter and notch filter were utilized to extract sEMG signals, while others recommended a Butterworth filter with specific parameters [27,28].

#### 2.2.2. Rectification

Given that sEMG signals fluctuate between −5 and 5 mV during muscle contraction [14,23], rectification is a critical preprocessing step, addressing the negative part of the signal. Two common approaches are full-wave rectification and half-wave rectification, with full-wave rectification typically being preferred due to its ability to represent the neural activation signal [29,30].

#### 2.2.3. Normalization

Since sEMG signals exhibit significant variability between individuals, amplitude normalization is essential for comparing signals across different subjects. Normalization involves dividing gathered sEMG signals by a reference sEMG value under identical conditions, facilitating inter-subject comparisons and enhancing computational efficiency [6,31].

#### 2.2.4. Segmentation

Segmentation divides the sampled data, post-preprocessing, into segments for subsequent feature extraction [32]. The size of the segments should be large enough to properly extract features from each segment and have a higher classification accuracy [33], but the length of these segments should also be small to avoid any computational delay in real-time systems. This was the motive for many studies to investigate the optimum window size for the sEMG signal [33,34]. The ideal controller delay for prosthetic controlling was found to be 100–125 ms [32]. As demonstrated in a previous study [35], a window size of 320 ms for prosthetic control was found to be imperceptible to users. Conversely, a recent investigation proposed an optimal window size in the range of 100–250 ms [36]. Our literature review leads to the conclusion that the ideal compromise between system delay and performance, whether using smaller or larger window sizes, strongly depends on the specific application.

There are two prevalent methods for segmenting sEMG signals: the adjacent windows method and the overlapping windows method. In the adjacent method, data are partitioned into predefined, non-overlapping segments, and features are extracted from each segment. However, this technique has the drawback of leaving the processor idle until the formation of the next segment. On the other hand, the overlapping windows method involves segments with overlap between each segment and its predecessor, facilitating the extraction of additional features [37]. Research has shown that overlapping windows tend to yield superior classification accuracy [33].

### 2.3. Feature Extraction

While classifiers can be trained using preprocessed raw signals, better accuracy is typically achieved by extracting features from these signals prior to model training [27,36,38]. Feature extraction not only enhances classifier performance but also reduces dimensionality, simplifying subsequent processing and classification [39]. Features can be classified into three categories: time domain features, frequency domain features, and time–frequency domain features [25], with classifiers often using a combination of features from these categories.

#### 2.3.1. Time Domain Features

Time domain features are evaluated based on signal amplitude variations over time, eliminating the need for further transformations and benefiting from their simplicity and low computational resource requirements [37]. A summary for the features is mentioned in Table A1.

#### 2.3.2. Frequency Domain Features

Frequency domain features, unlike time domain features, cannot be directly derived from raw data and are obtained by applying the Fourier transform to the signal. These features encompass the power spectrum density of the signal (PSD) [37]. A summary for the features is mentioned in Table A2.

#### 2.3.3. Time–Frequency Domain Features (TFD)

TFD combines time and frequency information, allowing the observation of different frequency components at various time intervals [37]. TFD proves especially valuable in capturing localized, transient, or intermittent components often overlooked by spectral-only methods like the FFT [40]. Various methods, such as the continuous wavelet transform (CWT) and discrete wavelet transform (DWT), are available for signal decomposition in the time–frequency plane, each offering unique advantages [41]. An array of techniques is available for signal decomposition in the time–frequency domain, each presenting distinct advantages. These methods encompass the Choi–William’s distribution (CWD), short-time Fourier transform (STFT), Wigner–Ville transform (WVT), and the CWT. Within the realm of time–frequency domain features, one notably effective approach is the wavelet transform (WT). According to [41], the WT predominantly comprises two distinct methods: the CWT and the DWT. Unlike the STFT, the WT is not confined to sinusoidal functions alone; it accommodates a wide array of waveforms, provided they meet predefined criteria. A summary for the features is mentioned in Table A3.

### 2.4. Classification and Evaluation

Several Machine Learning and deep learning approaches were employed for decoding sEMG signals, as summarized in Table 1.

## 3. Methods

**Figure 2:**
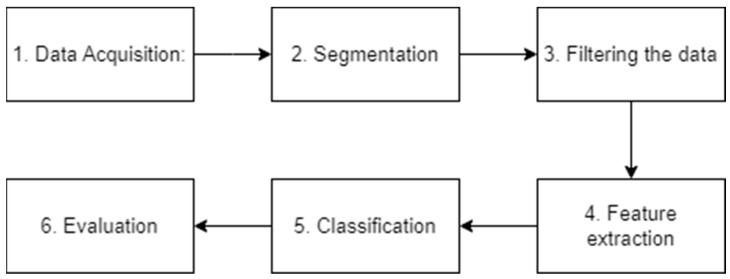
The proposed system block diagram.

After reviewing the previous work and analyzing their results, accordingly, our system block was designed as shown in Figure 2. The proposed system is formed from six steps in the same order as the block diagram. The system was designed so it can be used in real-time as the system is optimized for efficient operation on a microcontroller; this efficiency is obtained from the optimized Transformer architecture used for classification [46].

### 3.1. Data Acquisition

To procure our dataset, we opted for open-source resources that could fulfill our requirements sufficiently. We selected three different datasets, which are NinaPro (Non-Invasive Adaptive Prosthetics) Project’s NinaPro DB1, as made available through references [47,48]. Ninapro datasets were built to benchmark the sEMG-based gesture recognition algorithms. The dataset includes most of the movements used in everyday life, and rehabilitation exercises can be divided into three exercises: (1) basic finger movements; (2) isometric, isotonic hand configurations and wrist movements; and (3) grasping and functional movements.

Db-a and DB-b are sourced from CapgMyo [49]. These datasets encompass the surface sEMG recordings associated with eight distinct hand gestures executed by 18 and 20 individual subjects, respectively, with each gesture being captured in ten separate trials. The sEMG signals were meticulously sampled at a rate of 1000 Hz, ensuring high temporal resolution. The acquisition setup featured a set of sensors comprising eight electrode arrays, each measuring 8 units in width and 2 units in height. These electrode arrays were strategically affixed to the right forearm, forming an organized 8 × 16 grid configuration to capture the nuanced muscle activity patterns.

When constructing the Ninapro DB1 dataset, participants were instructed to pause for three seconds following each action. Consequently, the predominant class in the dataset became the resting motion, causing the number of samples for class zero to be twice that of any other class. This initial setup resulted in our experiment’s outcomes being overly tailored to class 0, which was deemed overfitting. To address this concern, we implemented a downsampling procedure aimed at reducing the number of instances in class zero (resting movement). This was achieved by retaining only the resting periods following the initial movement while removing subsequent rests after each movement.

### 3.2. Segmentation

Segmentation was executed by windowing the signals using a 320 ms window with a 100 ms overlap (equating to 32 samples per window with 10 overlapped samples). It was observed that increasing the number of samples within each segment positively impacted training accuracy. However, it is important to note that employing larger segments introduces delays in real-time systems. Thus, there exists a trade-off between achieving higher accuracy with larger window sizes and ensuring real-time performance in applications like prosthetic control.

### 3.3. Filtering the Data

Previous studies that utilized the same databases as our work have typically applied a Butterworth low-pass filter during signal preprocessing. Consistent with these prior approaches, we employed a similar filter for our data preprocessing [50,51,52].

### 3.4. Feature Extraction

A primary objective of our research is to explore and extract various features from the signals and employ them as input for the classifier to assess their impact on classification accuracy. Our approach involves extracting a single feature from each segment, followed by aggregating the segment values into a single value, thereby reducing the signal’s sample count. The features utilized in this work encompass (1) FFT and (2) wavelet transformation. These two features were identified as highly accurate in deep learning-based classification, as indicated by the findings in the existing literature [53,54,55]

#### 3.4.1. Fast Fourier Transformation [51]

For digital signals, the FFT facilitates the transformation of signals into the frequency domain, effectively determining the discrete Fourier transform of the input signal. The FFT computation is performed using a reduced set of mathematical equations, as expressed by the following formula:

$$F\left(K\Omega \right)=\sum_{n=0}^{N-1}f_{s}\left(nT\right)e^{-j(\frac{2\pi k_{n}}{N})}\;\;k=0,\;1,\;2\ldots \ldots N-1$$

where

- F(KΩ) is the discrete signal;
- N is the size of the domain.

#### 3.4.2. Wavelet Transformation [20]

When a wavelet transformation is applied to a signal, it undergoes decomposition into multiple “wavelets”, each characterized by distinct scales and positions of the primary function, known as the “mother wavelet”. Continuous wavelet transforms yield two coefficients: scale and frequency. The fundamental concept behind wavelet analysis involves expressing a signal as a linear combination of functions, which are obtained by shifting and dilating the mother wavelet. The continuous wavelet transformation of a continuous signal f(t) is mathematically defined as

$$c\left(a,b\right)=a\int -\infty +\infty s\left(t\right)\varphi \left(t-ba\right)dt,$$

where

- *a* is the scaling parameter, and *a* and *b* are the time-shift parameter;
- $\varphi \left(t-ba\right)$ is the mother wavelet function;
- $c\left(a,b\right)$ represents the wavelet coefficients.

In this study, we will focus on the Morlet and Mexican hat (Mexh) wavelet functions, which are among the most commonly employed wavelet transformations. These wavelet functions are defined as follows:

Morlet:

$$\psi \left(t\right)=e^{-\frac{t^{2}}{2}\cos\left(5t\right)}$$

Mexh:

$$\psi \left(t\right)=\frac{2}{\sqrt{3}\sqrt[{4}]{\pi }}e^{\frac{-t^{2}}{2}}\left(1-t^{2}\right)$$

where

- *t* is the time sequence.

### 3.5. Classification using ST

The initial step in our implementation process involves the creation of an image-shaped matrix derived from the sEMG signals subsequent to the feature extraction and normalization procedures. The formation of the image’s shape entails reshaping the 10 electrode readings from a 1D vector at time t (resulting in a 10 × 1 array) into a 2 × 5 matrix. To elaborate, the input signals to the classifier at time t are represented as

$$X\left(t\right)=[x_{1}\left(t\right),x_{2}\left(t\right),\ldots \ldots \ldots ..x_{10}\left(t\right)]$$

where

- *X*(*t*) is the input 1D vector to the classifier at time *t*;
- *X*_1_, *X*_2_ …… *X*_10_ are the output readings of each electrode at time *t* after the feature extraction step.

The input to the classifier assumes the following format:

$$X\left(t\right)=\left[\begin{array}{ccc} x_{1} & x_{2} & x_{3}\\ x_{6} & x_{7} & x_{8} \end{array}\begin{array}{cc} x_{4} & x_{5}\\ x_{9} & x_{10} \end{array}\right]$$

Following this, each resulting image adopts a final shape of (2 × 5). While several methods were explored for creating multi-layer matrix rather than using a 1 dimension matrix, such as retaining the electrode readings in the first channel and incorporating different features in each layer, no significant differences were observed in the final training accuracy. This matrix is aptly referred to as “Matrix signals”.

Subsequently, we performed data augmentation and normalization on the matrix signals. These signals underwent normalization and resizing, with additional data augmentations applied, including random flipping and rotation. Each matrix signal was resized to 72 × 72.

#### 3.5.1. ST Architecture Overview

**Figure 3:**
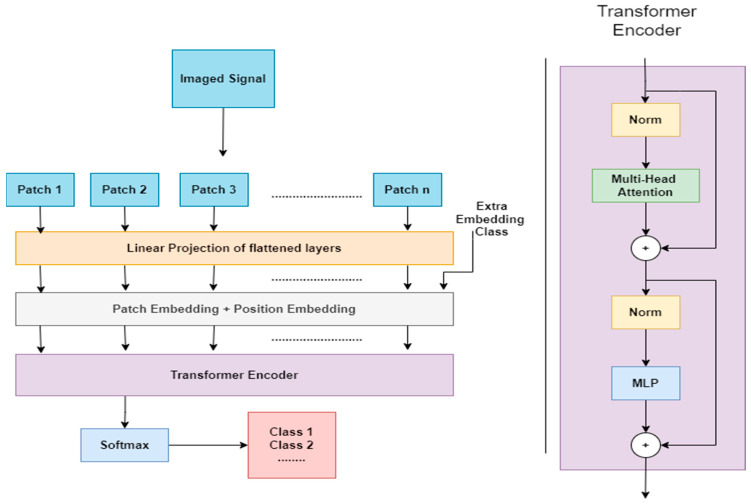
Model overview. Input patches are processed through linear projection, resulting in flattened patches that are transformed into embeddings. Position embeddings capture spatial information, and a CLS token is included for classification. The Transformer encoder head then processes these embeddings, followed by a softmax layer for classification.

Taking a top-down approach, we delve into the architecture of the ST, commencing with an overview of its structure and, subsequently, providing a detailed description of each component. An overview of the architecture is visually depicted in Figure 3. The architecture can be dissected into five key steps:

1. Split the matrix signals into patches;
2. Patch embeddings;
3. Position embeddings;
4. Transformer encoder;
5. Multilayer perceptron head.

##### Split the Matrix Signals into Patches

In order to adapt Transformers for processing 2D matrix signals, we first divide the matrix signals into distinct patches. For a matrix signal with the shape

$$x\in R^{H\times W\times C}$$

It shall be split into a sequence of 2D patches with shapes

$$x_{p}\in R^{P^{2}\times C},$$

where

- (*H*, *W*) is the resolution of the original image (height and width);
- *C* is the number of channels of the matrix (1 in our case);
- (*P*, *P*) is the resolution of the image patch.

The resulting number of patches will equal

$$N=HW/P^{2}$$

##### Patch Embeddings

The patches from the matrix signals, typically 16 × 16 in size, are then transformed into a D-dimensional vector using an embedding matrix E. This transformation aims to flatten the patches for compatibility with the Transformer, which only accepts a 1D input sequence of token embeddings.

##### Position Embeddings

In this step, the ST introduces the patch-embedded matrix as a class token (CLS token), instructing the model to classify the matrix signals. This forms an (N + 1) × D-dimensional vector, z. At the final classification step, the classification head is exclusively connected to the representation of the first token in the output of the final Transformer encoder head. This initial token serves as the image representation.

Additionally, position encoding is incorporated to indicate the original positional information of the patches within the original matrix signals. This enables differentiation between patches derived from various locations within the matrix signals. Importantly, the Transformer lacks inherent knowledge of the patch order, distinguishing it from Convolutional Neural Networks (CNNs). The combination of these two steps is represented as follows:

$$z_{0}=\left[x_{class};x_{p}^{1}E;x_{p}^{2}E;\ldots .;;x_{p}^{N}E\right]+E_{pos},E\in R^{{(P}^{2}.C)\times D},E_{pos}\in R^{\left(N+1\right)\times D}$$

where

- $z_{0}$ signifies the input sequence of embeddings for the Transformer encoder;
- $x_{class}$ is the prepended learnable class token;
- $x_{p}^{n}$ is the sequence of embedded patched;
- E is the embedding matrix;
- $E_{pos}$ is the position embedding.

##### Transformer Encoder

Our work employs the same Transformer encoder structure as utilized in [53], comprising alternating layers of multi-headed self-attention (MSA) and multi-layer perceptron (MLP). The configuration of the Transformer layers is articulated as follows:

$$z_{l}^{\prime }=MSA\left(LN\left(z_{l-1}\right)\right)+z_{l-1}\;\;1\;=\;1\ldots \ldots .\;L$$

$$z_{l}=MLP\left(LN\left(z_{l}^{\prime }\right)\right)+z_{l}^{\prime }\;\;\;1\;=\;1\ldots \ldots .\;L$$

where

- $z_{l}$ is the patch sequence representation output at layer l of the network;
- (*LN*) is the layer norm representation applied.

The patch sequence representation, denoted as $z_{l}$, traverses the Transformer block layers. In this process, it first undergoes layer normalization (LN), followed by multi-headed self-attention (MSA). Subsequently, a residual connection is introduced from the output representation of the preceding layer, $z_{l-1}.\;$Layer normalization is applied once more before feeding the sequence to the MLP. This multi−layer perceptron output is also coupled with the residual connection from the intermediate representation $z_{l}^{\prime }.$

##### Multilayer Perceptron Head (Classification Head)

The fifth and final step revolves around classification. The current work utilizes the first token, derived from the CLS token, from the output of the final Transformer layer ($z_{l}^{0}$). This token is directed to a feed-forward neural network (MLP) for the classification task. The construction of this step can be outlined as follows:

$$y=\mathrm{LN}\;(z_{l}^{0})$$

where

- *y* is the predicted class;
- $z_{l}^{0}$ is the first token of the Transformer’s final layer output.

### 3.6. Parameters Selection

Various parameters and hyperparameters required adjustment in our configuration process. These included determining the appropriate learning rate, specifying the number of Transformer heads for utilization, and opting for CWT as our method of choice. It is important to highlight that CWT exhibits significant variability based on the mother frequency employed; hence, we explored a range of mother frequencies to identify the most effective one. Additionally, when selecting the CWT, it is crucial to consider the scale, which, in the context of CWT, pertains to the measurement of how wavelets are stretched or compressed concerning their frequency and time domains.

Given our objective of establishing a single model applicable to all our datasets, we adopted a systematic approach to parameter and hyperparameter selection. Specifically, within the Ninapro DB-1 dataset, subjects 1, 7, and 22 were randomly chosen as representatives for this process. In the case of datasets, CapgMyo DB-A subjects 1 and 7 were similarly selected for parameter tuning, and a single subject 1 was chosen CapgMyo DB-B. Regarding the choice of mother frequency for the wavelet transformation, we considered two options: the Mexican hat and the Morlet Transform due to their established effectiveness and wide applicability in signal analysis. In the exploration of scales, we investigated three distinct ranges: scales ranging from 1 to 10, scales from 1 to 20, and scales spanning from 1 to 100. These deliberate selections were made to ensure a robust and adaptable model for our diverse datasets. The results are summarized in Table 2, Table 3, Table 4 and Table 5.

Hence, a learning rate of 0.0001 and 8 Transformer heads were selected, and the CWT will employ the Mexican hat as the mother frequency, with scales ranging from 0 to 10. The model’s hyperparameters are detailed in the following Table 6.

### 3.7. Evaluation

Based on the insights derived from our literature review, our research delves into the evaluation of classifiers, with a particular focus on inter-subject classification. In this context, we aim to assess the model’s performance using data from the different subjects and across different sessions, where electrodes are intentionally removed and subsequently reattached for each session.

To facilitate a meaningful comparison between our research and previous studies utilizing the NinaPro DB1 dataset, we adopt a consistent evaluation approach as employed in [50,51,52,56,57]. This evaluation method entails a 30–70 train–test split, albeit with specific criteria. Initially, a new model is initialized randomly for each subject, and training ensues on seven repetitions (i.e., repetitions 1, 3, 4, 6, 8, 9, and 10), followed by testing on three distinct repetitions (namely, repetitions 2, 5, and 7). The accuracy is computed for each individual subject, and subsequently, an average is calculated to derive the overall model accuracy.

For experiments conducted on the CapgMyo DB-a and DB-b datasets, we adhere to a training strategy akin to that described in [50,56]. Specifically, our model is trained on half of the available trials and subsequently tested on the remaining trials. This training methodology aligns with the approach of utilizing odd-numbered trials for model training and even-numbered trials for testing.

## 4. Results and Discussion

Three models were created for each dataset (in total, nine models). Table 7 summarizes the data for these models.

Afterward, all the models were evaluated on all the subjects for each dataset; then, the results were averaged to determine the final training accuracy, Macro F1 score, and Micro F1 score.

Accuracy and F1 (micro and macro) scores are chosen for model evaluation because they provide a comprehensive assessment of a model’s performance, especially in imbalanced datasets. Accuracy measures the overall correctness of the model, while F1 scores consider both precision and recall, which is crucial for models where false positives and negatives carry different costs. Micro F1 calculates metrics globally by counting the total true positives, false negatives, and false positives, ideal for balanced class distribution. Macro F1 averages the metrics for each class without considering class imbalance, highlighting performance in minority classes.

### 4.1. Training on NinaPro DB1

It was observed that training the model on the NinaPro DB1 suggests that the choice of feature extraction method can have a noticeable impact on model performance. While FFT showed a decrease in accuracy, CWT MEXH demonstrated a performance close to that of raw data, highlighting its potential for capturing relevant information with very low F1 Micro and Macro Scores. Results summary are found in Table 8.

### 4.2. Training on CapgMyo DB A

When training the model on the CapgMyo DB-A dataset, the FFT on the data slightly improved the accuracy to 74.90%, with the F1 Macro Score maintaining a similar level at 31.30%, while the F1 Micro Score remained at 70.00%. Interestingly, when Continuous Wavelet Transform with the Mexican hat wavelet applied as the feature extraction method, the accuracy showed a slight decrease to 72.90%. However, both the F1 Macro Score and F1 Micro Score experienced reductions, reaching 29.47% and 67.27%, respectively. Results summary are found in Table 9.

### 4.3. Training on CapgMyo DB B

Similarly, it can be observed that a marginal improvement in accuracy is achieved when applying the FFT as the feature extraction method for the input data. This slight increase in accuracy is accompanied by a modest rise in the F1 Macro score. However, it is noteworthy that the F1 Micro and Macro scores for this dataset remained relatively low. Results summary are found in Table 10.

Generally, the lower F1 Micro and Macro Scores can be attributed to several factors. First, the complexity of the gesture recognition task and the variability in hand movements across subjects may lead to challenges in achieving high precision and recall rates. Additionally, the relatively small size of the training dataset and potential class imbalance can impact the overall performance metrics. Furthermore, the choice of feature extraction method and model architecture can influence the model’s ability to capture subtle variations in the electromyographic signals associated with different hand gestures.

The notable disparity in accuracy between the FFT-based feature extraction method when applied to the CapgMyo datasets (DB-A and DB-B) versus the NinaPro DB1 dataset can be attributed to the sampling rate at which the EMG data was recorded. It was discovered that the NinaPro DB1 dataset was captured at a significantly lower sampling rate of 100 Hz. This sampling rate is considerably below the recommended frequency range for sEMG signals, which typically falls within the range of 5–500 Hz, necessitating a sampling frequency of 1000 Hz or higher for accurate signal representation. The use of dry electrodes, known to be less accurate and susceptible to motion artifacts compared to gel-based electrodes, further exacerbated the data quality issue in the NinaPro dataset. Consequently, the inadequate sampling rate and potential information loss in capturing EMG signals played a crucial role in the observed reduction in accuracy when applying FFT to the NinaPro DB1 dataset. In contrast, the CapgMyo datasets were recorded at the optimal sampling rate of 1000 Hz, resulting in more accurate and complete signal representation, which likely contributed to the improved accuracy observed when using FFT for feature extraction in these datasets.

### 4.4. Compared to Previous Work

For evaluating the model, various evaluation techniques were identified, including inter-subject and inter-session assessments. The inter-subject evaluation focuses on the performance of models across different subjects. This approach captures the variability inherent among various subjects, making it ideal for assessing the generalizability of a model. On the other hand, inter-session evaluation deals with the model’s performance across multiple sessions for the same subject. It often results in higher accuracy due to the consistency of the subject’s data but may lack generalizability [27]. The present work will focus on inter-subject evaluation, this method is crucial for determining the model’s generalizability, as it encompasses the variability inherent among different subjects. The Table 11 show a comparison between previous works with different evaluation methods.

In the case of NinaPro DB1 and CapgMyo DB-A, a comparison with prior studies reveals that the proposed approach excels over most models that adopted a similar strategy (the models with higher accuracy are not evaluated in the same method). Notably, it surpasses all other models that utilize the same strategy, except for one particular method. This observation highlights the impressive performance of the signal Transformer in sEMG signal classification tasks, even though the existing literature tends to emphasize the high accuracy achieved by CNNs. However, it is worth noting that when comparing results with CapgMyo DB-B [58], the previously mentioned method still outperforms the proposed approach.

When analyzing the internal representations of the Signal Transformer, it could also be applied to the presented matrix signals topology. The first layer of the Signal Transformer performs a linear projection of flattened patches into a lower-dimensional space. The learned embedding filters exhibit plausible basis functions for representing fine structures within each patch. Position embeddings are then added to the patch representations, encoding distance and capturing the row–column structure in the matrix signals. The position embeddings effectively represent 2D matrix signal topology, explaining why hand-crafted 2D-aware embedding variants do not yield improvements. The self-attention mechanism allows the Signal Transformer to integrate information across the entire matrix signal, even in the lower layers. Some attention heads attend to most of the image in the early layers, demonstrating the model’s ability to integrate information globally. In other terms, it gives insight for localizing the space of interest in the matrix signals (attention distance), i.e., which electrode readings affect the classification more than the other electrode readings. The attention distance increases with network depth, and the model attends to semantically relevant image regions for classification.

## 5. Conclusions

This study marks a significant advancement in the domain of sEMG signal recognition by pioneering the use of Signal Transformers, diverging from the conventionally favored convolutional neural networks (CNNs). By ingeniously converting sEMG signals into image-shaped matrices, we capitalized on the robust capabilities of standard Transformer encoders, predominantly used in natural language processing. This innovative approach not only enhanced the recognition process but also introduced a versatile methodology adaptable to various signal types.

Our findings compellingly demonstrate that the Novel Signal Transformer consistently outperforms most existing CNN architectures in sEMG signal classification. This superior performance is attributed to its ability to meticulously adapt to the matrix signals’ topology, an aspect where traditional CNN architectures lag. The initial layer’s adept linear projection captures the intricate structures within patches, while the strategic addition of position embeddings intricately maps the 2D matrix signals topology. Notably, the simplicity of this method outshone more complex, hand-crafted 2D-aware embedding variants, underscoring the elegance and effectiveness of the ST approach. A standout feature of the Signal Transformer is its self-attention mechanism, which facilitates a comprehensive integration of information across the spectrum, even in the initial layers. This mechanism is adept at discerning and focusing on the most pertinent regions within the matrix signals, thereby determining the influence of specific electrode readings on the classification outcome. As the network delves deeper, the attention span broadens, ensuring that the model remains attuned to semantically relevant regions for a more accurate and nuanced classification.

These findings not only challenge the prevailing biases favoring CNNs but also open up a plethora of possibilities for sEMG signal analysis and other related applications. They pave the way for further exploration and refinement of Transformer-based models in signal processing. Looking ahead, we envision extending this innovative approach to a wider array of signal types and classification tasks, potentially revolutionizing the way we interpret complex biological signals and their applications in medical technology and beyond.

## Figures and Tables

**Table 1:** Summary of some recent work applying ML and DL for decoding sEMG signals.

Title	Dataset	Subject/ Sessions (Total)	Classes/Channels	Time Window	Feature	Classifier	Accuracy (%)
[36]	NinaPro Database (DB) 5	10/6	18/8	260 ms	CWT	Transfer learning (TL) + CNN	68.98
[40]	Private	7/10	5/8	Not mentioned	Raw	CNN-LSTM	92.7
[42]	NinaPro DB1	27/10	52/10	2500 ms	Root Mean Square (RMS)	TCN	89.76/NA
[43]	NinaPro DB1-DB5-private	27/10-8/5-8/5	52/10-12/8-12/8	150ms-na-na	Non	CNN	71.85-55.31-78.98
[38]	DB 2, 3 and 4	40-11-10/6	17/12-17/12-12/12	200 ms	18 feature-Raw	TL + MLP-TL + CNN	67.00-68.00
[27]	DB1	27/10	52/8 × 16	40 frames centred	Raw + AdaBN	TL + ensemble CNN	56.50-67.40-Na
[44]	DB2	40/6	49/12	200 ms	Time + Frequency	CViT	80.02
[45]	CapgMyo Db A	18/8	8/128	100 ms	CNN	CNN + LSTM + TL	94.57

**Table 2:** Learning rate variations and performance metrics for different datasets and subjects (highest in bold).

Models	Accuracy for Learning Rate of 0.001	Accuracy for Learning Rate of 0.0001	Accuracy for Learning Rate of 0.00001
NinaPro DB1-subject 1	88.13	**89.85**	85.54
NinaPro DB1-subject 7	87.19	**88.52**	84.61
NinaPro DB1-subject 22	88.15	**89.85**	86.71
CapgMyo-A-subject 1	92.56	94.56	**94.61**
CapgMyo-A-subject 7	78.9	**78.39**	78.22
CapgMyo-B-subject 1	88.21	**89.1**	88.8
Average:	76.69	**77.87**	75.91

**Table 3:** Number of transformer heads variations and performance metrics for different datasets and subjects (highest in bold).

Models	Accuracy for 4 Transformer Heads	Accuracy for 8 Transformer Heads	Accuracy for 16 Transformer Heads
Nina-subject 1	**90.34**	89.68	90.13
Nina-subject 7	87.56	87.6	**87.8**
Nina-subject 22	91.99	**92.43**	91.77
CapgMyo-A-subject 1	94.22	**94.61**	92.83
CapgMyo-A-subject 7	77.83	78.22	**80.06**
CapgMyo-B-subject 1	88.8	**89.1**	87.4
**Average:**	78.29	**78.44**	76.49

**Table 4:** Morlet wavelet parameters and performance metrics for different scales, datasets and subjects (highest in bold).

Models	Accuracy for Morl Scale 1–10	Accuracy for Morl Scale 1–20	Accuracy for Morl Scale 1–100
Nina-subject 1	89.09	**89.22**	89.18
Nina-subject 7	88.31	**88.42**	87.41
Nina-subject 22	91.99	**92.39**	91.95
CapgMyo- A-subject 1	94.17	94.11	**94.28**
CapgMyo- A-subject 7	78	78.67	**78.94**
CapgMyo- B-subject 1	**88.28**	88.11	87.94
**Average**	78.3	**78.48**	78.28

**Table 5:** Mexican hat wavelet parameters and performance metrics for different scales, datasets and subjects (highest in bold).

Models	Accuracy for Mexh Scale 10	Accuracy for Mexh Scale 20	Accuracy for Mexh Scale 100
Nina-subject 1	89.38	**88.64**	**88.64**
Nina-subject 7	**88.46**	87.6	87.84
Nina-subject 22	91.84	**92.02**	91.99
CapgMyo-A-subject 1	73.17	74.83	**75.33**
CapgMyo-A-subject 7	**81.00**	77.72	79.00
CapgMyo-B-subject 1	89.78	**80.11**	89.56
**Average**	**78.93**	78.48	78.72

**Table 6:** Hyperparameters used for the training.

Batch Size	Matrix Signal Size	Num of Epochs	Eval Steps	Learning Rate	Weight Decay	Transformer Layers	Input Patch Size	MLP Head Size
55	72 × 72	8	100	0.0001	0.0001	8	6	2048 × 1024

**Table 7:** Summary of the models that were training.

Model Number	Data Set	Feature Extracted	Model Name
1	Ninapro DB1	Raw Data	ST-Nina-RAW
2	Ninapro DB1	Fast Fourier Transform	ST-Nina-FFT
3	Ninapro DB1	CWT—Mexican hat	ST-Nina-MEXH
4	CapgMyo DB A	Raw Data	ST-Capg-A-RAW
5	CapgMyo DB A	Fast Fourier Transform	ST-Capg-A-FFT
6	CapgMyo DB A	CWT—Mexican hat	ST-Capg-A-MEXH
7	CapgMyo DB B	Raw Data	ST-Capg-B-RAW
8	CapgMyo DB B	Fast Fourier Transform	ST-Capg-B-FFT
9	CapgMyo DB B	CWT—Mexican hat	ST-Capg-B-MEXH

**Table 8:** Summary of the results of the three models for NinaPro DB1.

Model Name	Accuracy (%)	F1 Macro Score (%)	F1 Micro Score (%)
ST-Nina-RAW	**85.97**	**14.25**	57.90
ST-Nina-FFT	85.30	13.27	**61.74**
ST-Nina-MEXH	85.92	14.14	58.88

**Table 9:** Summary of the results of the three models for CapgMyoDB A.

Model Name	Accuracy (%)	F1 Macro Score (%)	F1 Micro Score (%)
ST-Capg-A-RAW	77.79	30.70	**70.60**
ST-Capg-A-FFT	**79.90**	**31.30**	70.00
ST-Capg-A-MEXH	77.90	29.47	67.27

**Table 10:** Summary of the results of the three models for CapgMyoDB B.

Model Name	Accuracy (%)	F1 Macro Score (%)	F1 Micro Score (%)
ST-Capg-B-RAW	71.36	25.59	58.6
ST-Capg-B-FFT	**72.92**	**27.00**	**61.96**
ST-Capg-B-MEXH	71.57	25.94	58.36

**Table 11:** Summary of previous work that used the same DB (CapgMyo DB A) and same evaluation method.

Reference	Database	Algorithm	Accuracy	Evaluation Method
[58]	Ninapro DB1	Self-Learning	40.24	Inter-subject
[59]	Ninapro DB1	SVM	75.2%	Inter-subject
[57]	Ninapro DB1	CNN	78.75%	Inter-subject
[52]	Ninapro DB1	Random Forest	75.32%	Inter-subject
[52]	Ninapro DB1	CNN	66.60%	Inter-subject
[56]	Ninapro DB1	CNN	78.90%	Inter-subject
[51]	Ninapro DB1	CNN	70.48%	Inter-subject
[50]	Ninapro DB1	CNN	85.00%	Inter-subject
[60]	Ninapro DB1	CNN	85.50%	Inter-subject
[61]	Ninapro DB1	CNN + Attention	86.00%	Inter-Session
[62]	Ninapro DB1	CNN	91.40%	Inter-Session
[58]	CapgMyo A	Self-Learning	76.31%	Inter-subject
[63]	CapgMyo A	Linear Regression	77.57%	Inter-subject
[63]	CapgMyo A	Position weight	75.00%	Inter-subject
[64]	CapgMyo A	MLP	90.50%	Inter-Session
[58]	CapgMyo B	Self-Learning	79.86%	Inter-subject
[59]	CapgMyo B	SVM	75.40%	Inter-subject
[64]	CapgMyo B	MLP	90.30%	Inter-Session

## Data Availability

Data are contained within the article.

## Appendix A

List of the time domain features:

There exists a collection of over 27 time domain features, and Table A1 provides a concise overview of a subset of these features. The associated formulas are derived by partitioning the signal x into windows of length L, where x_i,k_ denotes the kth element within the ith window.

**Table A1:** Summary of the time domain features.

Feature	Formula	Explanation
Mean Absolute Value (MAV) [65]	MAV (xi) = $\frac{1}{L}\sum_{k=1}^{L}\left|x_{i,k}\right|$	The moving average of the signal.
Waveform length (WL) [66]	$\mathrm{WL}\;(\mathrm{xi})=\sum_{k=1}^{L}\left|x_{i,k}-x_{i,k-1}\right|$	Offers a simple characterization of the amplitude, duration, and frequency of the signal.
Zero Crossing (ZC) [36]	$\{x_{i,k}>0\;and\;x_{i,k+1}<0$ $\}\;\mathrm{or}\;\{x_{i,k}>0\;and\;x_{k+1}<0$ $\}\;\mathrm{and}\;\left|x_{k}-x_{k+1}\right|\geq \epsilon$	Counts the frequencies at which the signal passes through zero (a threshold ∈>0 is used to avoid noise).
Variance (VAR) [24]	$VAR=\frac{1}{L}\sum_{k=1}^{L}x_{i,k}^{2}$	An index to the power of the signal.
Root Mean Square (RMS)	$RMS\left(xi\right)=\sqrt{\frac{1}{L}\sum_{k=1}^{L}x_{i,k}^{2}}$	Also known as the quadratic mean. Related to the standard deviation when the mean of the signal = 0.
Average Amplitude Change (AAC) [36]	$AAC=\frac{1}{L}\sum_{k=1}^{L}\left|x_{i,k+1}-x_{i,k}\right|$	Shows the mean value by which the amplitude of the signal changes
Slope sign change (SSC) [36]	ssc= ${(x}_{i,k}-x_{i,k-1})*{(x}_{i,k}-x_{i,k+1})\geq \epsilon$	Measures the frequency at which the signal changes the slope sign (derivative).
Skewness (SKEW) [36]	$\mathrm{SKEW}=\frac{\sum_{k=1}^{L}{{(x}_{i,k}-{\bar{x}}_{i})}^{3}}{L*\sigma^{3}}$ Where σ is the standard deviation	Measures the asymmetry of the distribution.
Autoregressive coefficient (AR) [36]	$x_{i,k}=\sum_{j=1}^{P}\rho_{j}x_{i,k-j}+\epsilon_{t}$ $\mathrm{Where}\;p\;\mathrm{is}\;\mathrm{the}\;\mathrm{model}\;\mathrm{order}\;\mathrm{and}\;\rho_{j}$$\;\mathrm{is}\;\mathrm{the}\;j^{th}$ $\mathrm{coefficient}\;\mathrm{of}\;\mathrm{the}\;\mathrm{model}\;\mathrm{and}\;\epsilon_{t}$ is the residual noise	Aims to predict the future values of the signal based on the weighted average of the previous data. It shows each sample point as a linear combination of previous samples and an error.
Integrated EMG (IEMG)	$IEMG\left(xi\right)=\sum_{k=1}^{L}\left|x_{i,k}\right|$	Returns the absolute sum of the segment.
Myopulse Percentage Rate (MYOP)	$MYOP=\frac{l}{L}\sum_{K=1}^{L}if(\left|x_{i}\right|>threshold)$	Shows the mean absolute value of the segment of the windows that is larger than an amplitude threshold value.
Temporal Moment (TM)	$TM=\left|\frac{1}{L}\sum_{K=1}^{L}x_{i,k}^{order}\right|$	The 1st order is the MAV, and the 2nd order is the variance; thus, it usually starts from the 3rd order. It is a statistical analysis technique that can also be used as a feature.
V-order (VO) [67]	$VO={\left(\frac{1}{L}\sum_{K=1}^{L}x_{i}^{order}\right)}^{\frac{1}{order}}$	According to [6] it gives an insight into the force of muscle contraction.
Mean Absolute Derivative (MAD)	$MAD=\frac{1}{L}\sum_{k=1}^{L}\left|x_{i,k}-{\bar{x}}_{i}\right|$	Shows the distance between each sample of the window and the mean.

where *x_i_*: Represents the signal or a specific sample (*i*) in the signal; *L*: Denotes the length of the signal or the number of samples in the signal; *K*: Indicating the index of the current sample in the signal; *ρ*: Standard deviation of the signal.

List of the frequency domain features:

The following table provides a condensed summary sourced from [67] of several of these features. The formulas are computed by segmenting the signal x into windows of length L, with xi,j representing the jth element within the ith window. These calculations involve the signal’s frequency f and power spectrum p.

**Table A2:** Summary of the frequency domain features.

Feature	Formula	Explanation
Mean frequency (MNF)	$MNF=\frac{\sum_{j=1}^{M}f_{i,j}P_{i,j}}{\sum_{k=1}^{L}\left|x_{i,j}\right|}$	The average frequency.
Median frequency (MDF)	$\sum_{j=1}^{MDF}P_{i,j}=\sum_{j=MDF}^{M}P_{i,j}=\frac{1}{2}\sum_{j=1}^{M}P_{i,j}$	The frequency divides the spectrum into two regions that are equal in amplitude.
Mean power frequency (MNP)	$MNP=\frac{\sum_{j=1}^{M}P_{i,j}}{M}$	The average power of the power spectrum.
Peak frequency (PF)	$\mathrm{PKF}=\max\;(P_{i,j}$) where j=1, 2 …….M.	The frequency corresponds to the highest power.
Total power (TTP)	$TTP=\sum_{j=1}^{M}P_{i,j}$	A summation of the sEMG power spectrum.
Frequency ratio (FR)	$FR=\frac{\sum_{j=LLC}^{ULC}P_{i,j}}{\sum_{j=LHC}^{UHC}P_{i,j}}$ where *ULC* and *LLC* are the upper- and lower-cutoff frequency of the low-frequency band, and *UHC* and *LHC* are the upper- and lower-cutoff frequency of the high-frequency band, respectively.	The ratio between the highest and lowest frequency components of the sEMG signals used to distinguish between the contraction and relaxation of the muscles.
Power spectrum ratio (PSR)	$PSR=\frac{P_{0}}{P}=\frac{\sum_{j=f_{o-n}}^{f_{o}+n}P_{i,j}}{\sum_{j=-\infty }^{\infty }P_{i,j}}$ $\mathrm{Where}\;f_{0}$ is a feature value of the FPK, and *n* is the limit for integration	The ratio between the energy (nearly the maximum value of the sEMG power spectrum) and the energy P, which is the whole energy of the sEMG power spectrum.

List of the time–frequency domain features [20]:

A selection of time–frequency features is presented in the table below.

**Table A3:** Summary of the time–frequency domain features.

Feature	Formula	Explanation
Continuous Wavelet transform (CWT)	$CWT(a,b)=\frac{1}{\sqrt{a}}\int_{-\infty }^{+\infty }x\left(t\right)\psi^{*}\left(\frac{t-b}{a}\right)dt$ where $\psi \;\left(t\right)\;is\;the\;mother\;wavelet,$ *a* is a scale parameter and *b* is a translation parameter	Uses every possible wavelet in a range of locations and scales through the changing parameters and b.
Discrete Wavelet Transformation (DWT)	$DWT\left(n,m\right)=\int_{-\infty }^{+\infty }x\left(t\right)\psi_{n,m}\left(t\right)dt$ and $\psi_{n,m}\left(t\right)=\frac{1}{\sqrt{a_{0}^{m}}}\psi (\frac{t-nb_{0}a_{0}^{m}}{a_{0}^{m}})$ where *m* is the dilation parameter, *n* is the translation parameter, and $a_{0}$ is the step parameter	Uses defined wavelets in a range of locations and scales.
